# First Report of Skunk Amdoparvovirus (Species *Carnivore amdoparvovirus 4*) in Europe in a Captive Striped Skunk (*Mephitis mephitis*)

**DOI:** 10.3390/v15051087

**Published:** 2023-04-28

**Authors:** Franziska K. Kaiser, Madeleine de le Roi, Wendy K. Jo, Ingo Gerhauser, Viktor Molnár, Albert D. M. E. Osterhaus, Wolfgang Baumgärtner, Martin Ludlow

**Affiliations:** 1 Research Center for Emerging Infections and Zoonoses, University of Veterinary Medicine Hannover, Foundation, 30559 Hannover, Germany; 2 Department of Pathology, University of Veterinary Medicine Hannover, Foundation, 30559 Hannover, Germany; 3 Hannover Adventure Zoo, 30175 Hannover, Germany

**Keywords:** skunk amdoparvovirus, parvovirus, striped skunk, next generation sequencing, virus surveillance

## Abstract

Skunk amdoparvovirus (*Carnivore amdoparvovirus 4*, SKAV) is closely related to Aleutian mink disease virus (AMDV) and circulates primarily in striped skunks (*Mephitis mephitis*) in North America. SKAV poses a threat to mustelid species due to reported isolated infections of captive American mink (*Neovison vison*) in British Columbia, Canada. We detected SKAV in a captive striped skunk in a German zoo by metagenomic sequencing. The pathological findings are dominated by lymphoplasmacellular inflammation and reveal similarities to its relative *Carnivore amdoparvovirus 1*, the causative agent of Aleutian mink disease. Phylogenetic analysis of the whole genome demonstrated 94.80% nucleotide sequence identity to a sequence from Ontario, Canada. This study is the first case description of a SKAV infection outside of North America.

## 1. Introduction

The genus *Amdoparvovirus* in the family *Parvoviridae* is currently comprised of five species each containing a single virus, *Carnivore amdoparvovirus 1* (Aleutian mink disease virus, AMDV), *Carnivore amdoparvovirus 2* (gray fox amdovirus, GFAV), *Carnivore amdoparvovirus 3* (raccoon dog and fox amdoparvovirus, RFAV), *Carnivore amdoparvovirus 4* (skunk amdoparvovirus, SKAV), and *Carnivore amdoparvovirus 5* (red panda amdoparvovirus, RpAPV) [1,2]. Amdoparvoviruses are small, non-enveloped virus particles that contain a single-stranded, negative-sense DNA genome of approximately 4.8 kb [3,4]. This contains two gene cassettes, which encode for multiple proteins via alternative splicing for the non-structural proteins NS1, NS2, NS3, and the more conserved capsid proteins VP1 and VP2, respectively [5]. Amdoparvoviruses are known for genetic plasticity with frequent recombination events [6] and a wide host range within the order *Carnivora* including different mink species (*Mustela* spp.), arctic, gray, and red foxes (*Vulpes* spp.), lynx (*Lynx rufus*), raccoon dogs (*Nyctereutes procyonoides*), red panda (*Ailurus fulgens*), otters (*Lutrinae*), badgers (*Meles meles*), and marten species (*Martes* spp.) [7,8,9,10,11,12].

The prototypical representative of this genus, AMDV can cause high economic losses in mink farms [13] and has also been associated with rare diseases in humans [14,15]. It is assumed that AMDV originated in North America and was transported to Europe as a result of transcontinental animal trading [16,17]. Genomic investigations have demonstrated transmission of AMDV between wild and farmed mink and have highlighted the role of wild animals as a possible long-term virus reservoir [18,19]. The severity of amdoparvovirus-associated disease ranges from subclinical, persistent infections in healthy carriers to lethal systemic disease, depending on the virus strain and host species [4,20]. A fatal outcome is particularly noted in farmed mink possessing the Aleutian coat color gene, responsible for grey fur, upon infection by AMDV.

Skunk amdoparvovirus (SKAV) is widespread in North America, with an unknown genetic diversity [21]. SKAV infection has been reported in free-ranging skunks (*Mephitis mephitis*) in British Columbia and California with an incidence of 86% and 65%, respectively [22,23], and in a captive skunk [24]. SKAV is not host restricted with infection also reported in mink and ferrets (*Mustela putorius furo*) [16,25]. However, in common with other amdoparvoviruses, the potential of SKAV to infect different carnivore species remains largely uncharacterized. In general, the most common lesions observed in animals infected with amdoparvoviruses comprise splenomegaly and lymphoplasmacellular inflammation in various organ systems with reports of nephritis, myocarditis, encephalitis, and pneumonia [5,10,26,27].

In this study, we have identified the first case of SKAV infection in Europe in a captive striped skunk in Germany and have analyzed the pathology and evolutionary relationship of this strain to previously described amdoparvoviruses.

## 2. Materials and Methods

### 2.1. Gross Pathology and Histopathology

A 7-year-old male striped skunk was submitted to the Department of Pathology (University of Veterinary Medicine Hannover, Foundation) in 2016. Initially, the animal was in private possession in Germany (North Rhine-Westphalia) and subsequently transferred to a zoo in Germany (Lower Saxony), where it was kept until euthanasia. During a comprehensive post-mortem examination, organ samples were harvested and stored at −80 °C for molecular diagnostic assays, as well as in 10% neutral-buffered formalin for 24 h prior to embedding in paraffin wax for the purpose of histological investigations. Sections (2–4 µm) of formalin-fixed and paraffin-embedded (FFPE) tissues were mounted on SuperFrost^®^ Plus slides (Glasbearbeitungswerke GmbH & Co. KG, Braunschweig, Germany) and stained with hematoxylin and eosin. Furthermore, iron deposits (hemosiderin) and collagen fibers were detected on selected slides using Turnbull’s blue and Azan staining, respectively.

### 2.2. Genome Sequencing and Analysis

Spleen, liver, and kidney samples from a diseased striped skunk (sample number S521/16) were processed for metagenomic sequencing. Tissue preparation included three freeze/thaw cycles of homogenized sample material and OmniCleave endonuclease treatment (Epicenter Biotechnologies, Madison, WI, USA). Nucleic acids were extracted with a guanidinium thiocyanate-phenol-chloroform extraction using TRIzol (Qiagen, Hilden, Germany). Viral sequences were enriched within these samples by use of a sequence-independent single-primer amplification (SISPA) protocol [28] modified with non-ribosomal hexamers [29]. Preparation of cDNA libraries was achieved using a Nextera XT DNA Sample Preparation Kit (Illumina, San Diego, CA, USA) prior to sequencing on an Illumina MiSeq sequencer (MiSeq Reagent Kit v3, 2 × 300 cycles, Illumina, San Diego, CA, USA). For downstream analysis, raw reads were mapped against the SKAV reference genome (GenBank accession no. NC_034445.1) using Geneious Prime (Biomatters, Ltd., Auckland, New Zealand). In addition, we confirmed the exact sequence of a G-rich region of the SKAV genome between nucleotide positions 2503–2516 (GenBank accession no. OQ294046), by PCR using Phusion^®^ High-Fidelity PCR kit (NEB, Ipswich, MA, USA) with the forward primer 5′-GTTCCTCAGCACTATCCTG-3′ and reverse primer 5′-GTATCAGTAGTTCTACCAGC-3′ prior to Sanger sequencing. This region has a variable length in different published *Carnivore amdoparvovirus 4* genomes. The complete genome sequence of the SKAV strain was deposited on GenBank (GenBank accession no. OQ294046).

### 2.3. Phylogenetic Analysis

The evolutionary relationship between carnivore amdoparvovirus representatives was investigated for whole genome sequences, and NS1 and VP2 genes. A multiple sequence alignment of amdoparvoviruses was generated with sequences downloaded from GenBank using MAFFT version 7 [30]. The general time reversible model with a discrete gamma distribution (+G) and some evolutionarily invariable sites (+I) was calculated as the best fit with MEGA X for all alignments and used for the maximum likelihood method with 1000 bootstraps [31]. MEGA X was used to perform the analysis with 40 sequences each [32]. Branch lengths illustrate the number of nucleotide substitutions per site as indicated by the scale bar. Recombination analyses were performed with the integrated software package RDP4 [33], which included the algorithms RDP, GeneConv, Bootscan, MaxChi, Chimera, SiScan, and 3Seq with an initial cut-off *p*-value of 0.01. The criteria for selection of recombination events for further analyses was detection by at least three of the algorithms and with *p* < 0.05.

### 2.4. Statistics

For calculating the divergence between available complete NS1 and VP2 coding sequences of SKAV, means and standard deviation were calculated and a two samples t-test was conducted to test the null hypothesis μ1−μ2 = 0. The sample size for the comparison of 31 NS1 sequences was *n* = 465 and *n* = 528 for all 33 VP2 sequences.

## 3. Results

### 3.1. Macro- and Histopathology Findings

The skunk with a 2.09 kg body weight showed elevated liver and kidney parameters, mild ascites, and ataxia of the hind limbs and was euthanized due to poor general condition. Symptoms were observed for at least four months prior to euthanasia. Ultrasonographic and radiological examination revealed calcifications of the renal pelvis, thickened and calcified vessels of the liver as well as thickened bile ducts. At necropsy, the animal was in a good to moderate nutritional condition and showed severe jaundice. The amount of urea determined in the anterior eye chamber fluid exceeded the maximum detection limit and was greater than 300 mg/dL substantiating uremia [34]. The entire liver showed miliary yellowish-white to greyish-red nodules throughout the parenchyma and on the capsule. Further findings included a marked splenomegaly and an irregular surface of both kidneys accompanied by a striated cortex. The vessels in the renal pelvis, renal artery and vein, abdominal aorta, vena cava, and mesenteric and coronary vessels were markedly thickened, hardened, and whitish in color. Both parathyroid glands were mildly to moderately enlarged. The lung displayed multiple up to 5 mm in diameter, whitish, subpleural nodules.

**Figure 1:**
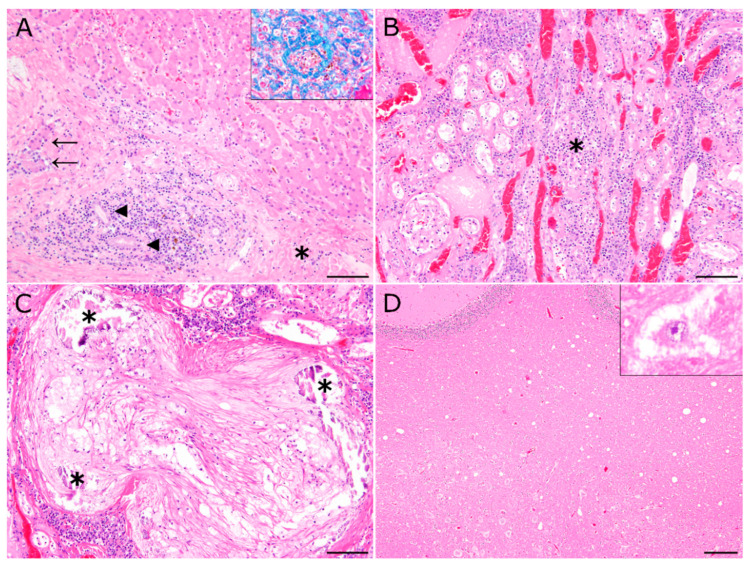
Histopathologic findings. (**A**) Mild lymphoplasmacellular inflammation, moderate fibrosis (asterisk), and mild biliary duct hyperplasia (arrowheads) in portal triads. Fibrosis extended into the adjacent parenchyma surrounding islands of intact hepatocytes (arrows, scale bar: 100 µm, hematoxylin and eosin). Insert: Fibrosis is characterized by increased numbers of bluish collagen fibers (Azan stain). (**B**) Severe multifocal to coalescing non-suppurative interstitial nephritis (asterisk) with moderate interstitial fibrosis (scale bar: 100 µm, hematoxylin and eosin). (**C**) Severe multifocal calcification (asterisks) of a renal vessel associated with granulomatous inflammation (scale bar: 100 µm, hematoxylin and eosin). (**D**) Mild diffuse vacuolization of the cerebellar white matter (scale bar: 200 µm, hematoxylin and eosin). Insert: Moderate vacuolization of cerebellar neurons (hematoxylin and eosin).

Histopathological lesions of the liver were characterized by severe inter- and intralobular septating fibrosis partly replacing the original parenchyma, dilated and multifocally capillarized sinusoids accompanied by an intrasinusoidal fibrosis, a mild lymphoplasmacellular portal inflammation, a mild biliary duct hyperplasia and a mild multifocal hemosiderin deposition (Figure 1A). Both kidneys showed severe multifocal to coalescing non-suppurative interstitial nephritis with moderate interstitial fibrosis (Figure 1B). The tunica media and tunica adventitia of the abdominal aorta, vena cava, coronary arteries, mesenteric blood vessels, serosal blood vessels of the urinary bladder and colon as well as blood vessels of the kidney and tongue showed a severe multifocal to coalescing calcification associated with granulomatous inflammation and occasionally cholesterol crystal deposition (Figure 1C). In addition, calcifications were also noted within the gastric submucosa. Furthermore, mild to moderate multifocal lymphoplasmacellular gastroenteritis was observed. Central nervous system lesions included a mild multifocal vacuolization of the subcortical white matter and moderate meningeal fibrosis with mild calcifications in the cerebrum as well as a mild diffuse vacuolization of cerebellar white matter accompanied by moderate vacuolization of cerebellar neurons (Figure 1D). A mild gliosis, several dilated myelin sheaths with myelinophages, and few spheroids were detected in the cervical and thoracic spinal cord. The lumbar spinal cord displayed mild neuronal vacuolization and moderate lymphoplasmacellular meningitis. Additional lesions comprised mild to moderate lymphoplasmacellular to suppurative rhinitis and a severe vacuolization of the adrenal cortex. The pulmonary and mesenteric lymph nodes showed mild to moderate follicular hyperplasia and moderate plasmocytosis. The spleen was characterized by marked extramedullary hematopoiesis and mild to moderate hyperplasia of both red and white pulp. Multiple foam cell granulomas, atelectasis, alveolar edema, and mild hemorrhages were found in the lung.

### 3.2. Virus Detection and Phylogenetic Analysis

**Figure 2:**
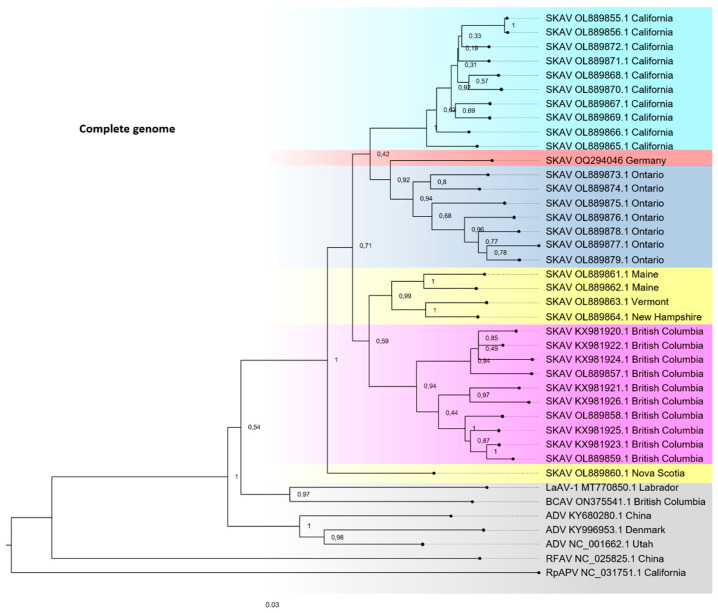
Evolutionary analysis of different amdoparvoviruses based on the complete genome sequences. SKAV genomes from similar geographical areas are highlighted in the same color and SKAV-related amdoparvoviruses are highlighted in grey. A total of 1000 bootstraps, GTR + G + I; +G, parameter = 0.3359; +I, 31.80% sites; log likelihood of presented tree −32,286.07. ADV, Aleutian mink disease parvovirus (*Carnivore amdoparvovirus 1*); BCAV, British Columbia amdoparvovirus (unclassified); SKAV, skunk amdoparvovirus (*Carnivore amdoparvovirus 4*); LaAV-1, Labrador amdoparvovirus 1 (*Carnivore amdoparvovirus 6*); RFAV, racoon dog and fox amdoparvovirus (*Carnivore amdoparvovirus 3*); RpAPV, red panda amdoparvovirus (*Carnivore amdoparvovirus 5*).

**Figure 3:**
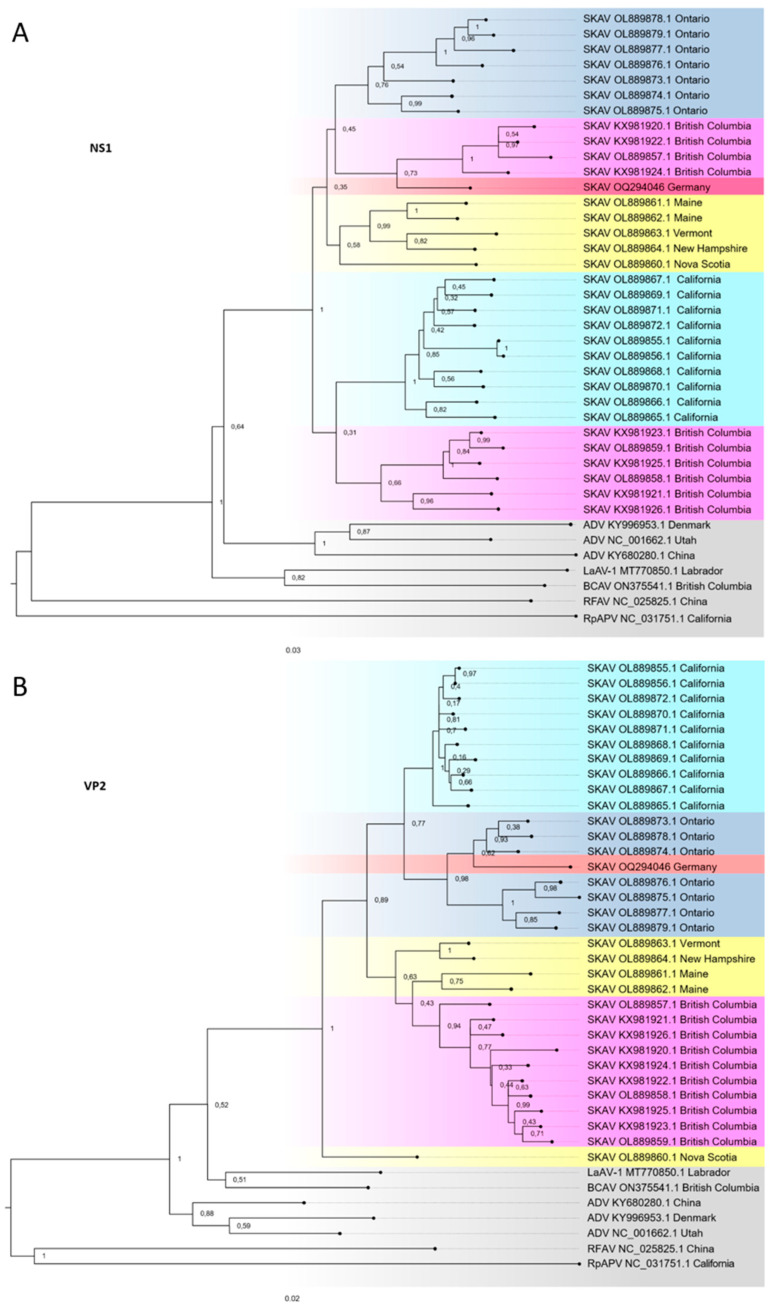
Phylogenetic analysis of the German SKAV strain. (**A**) Maximum likelihood tree based on forty complete 2008 bp NS1 nucleotide sequences. A total of 1000 bootstraps, GTR + G + I; +G, parameter = 0.5105); +I, 26.63% sites; log likelihood of presented tree −17,203.40. (**B**) Maximum likelihood tree based on forty complete 1941 bp VP2 nucleotide sequences (1000 bootstraps, GTR + G + I; +G, parameter = 0.2312; +I, 35.66% sites; log likelihood of presented tree −10,250.79. (**A**,**B**) SKAV genomes from similar geographical areas are highlighted in the same color and SKAV-related amdoparvoviruses are highlighted in grey. ADV, Aleutian mink disease parvovirus (*Carnivore amdoparvovirus 1*); BCAV, British Columbia amdoparvovirus (unclassified); SKAV, skunk amdoparvovirus (*Carnivore amdoparvovirus 4*); LaAV-1, Labrador amdoparvovirus 1 (*Carnivore amdoparvovirus 6*); RFAV, racoon dog and fox amdoparvovirus (*Carnivore amdoparvovirus 3*); RpAPV, red panda amdoparvovirus (*Carnivore amdoparvovirus 5*).

Given that the pathological analyses of tissues from the skunk were indicative of a chronic carnivore amdoparvovirus virus infection, and the well-documented sequence diversity of members of the genus Amdoparvovirus, next-generation sequencing (NGS) was performed to confirm this hypothesis. Mapping of recovered reads to a reference *Carnivore amdoparvovirus 4* sequence derived from a skunk (GenBank accession no. NC_034445.1) enabled the assembly of a 4583 bp genome, which contained an additional 166 bp and 172 bp at the 5′ and 3′ genomic ends, respectively, compared to the reference sequence. Phylogenetic analyses based on whole genome sequences and NS1 and VP2 coding sequences were performed to determine the evolutionary relationship of this SKAV genome sequence to other SKAV strains. Based on analysis of the complete genome sequence, the German SKAV strain groups with sequences from Ontario, Canada (Figure 2). It is most closely related to a strain characterized in 2022 from Ontario (OL889876.1), sharing 94.80% nucleotide identity. Due to frequently observed recombination events among amdoparvoviruses and associated differential evolutionary dynamics in both ORFs, the phylogenetic relationships of both NS1 and VP2 sequences were analyzed separately. A sequence identity of 94.82% was observed in the NS1 coding sequence with a SKAV strain from British Columbia, Canada in 2022 (GenBank accession no. OL889857.1) (Figure 3A). The highest nucleotide identity of the VP2 coding sequence was with a strain also characterized in 2022 from Ontario, Canada (GenBank accession no. OL889876.1) (Figure 3B). Given the discordant geographical clustering displayed by the German SKAV strain in the phylogenetic trees constructed using NS1 and VP2 sequences, recombination analysis was performed using the software package RDP4 on full-length SKAV genome sequences. However, no evidence could be detected of a potential recombination event in the German SKAV genome sequence, which met the criteria for a positive event. A comparison of sequence similarity from all available VP2 coding sequences revealed an average nucleotide sequence identity of 95.16% (1.82 SE). Nucleotide sequences of NS1 coding sequences share an average nucleotide similarity of 92.60% (1.56 SE). This demonstrates a significantly (*p* < 0.05) higher degree of conservation on the nucleotide level of VP2 coding sequences compared to NS1 coding sequences.

## 4. Discussion

Skunk amdoparvovirus (species *Carnivore amdoparvovirus 4*; SKAV) is endemic in North American skunk populations. However, little is known about the prevalence and sequence diversity of SKAV outside of Canada and the USA. In this study, we have pathologically and molecularly characterized a case of SKAV infection in a captive striped skunk in Germany. Although this case represents the first description of SKAV outside of North America, the German strain was found to be closely related to a clade of SKAV sequences identified previously in Ontario, Canada. The animal investigated in this study had resided in a zoo in Lower Saxony, Germany, and was previously in private possession in Germany, but further epidemiological information concerning the origin, travel history, and contact species was unfortunately unavailable. However, the phylogenetic analyses suggest that this virus strain was introduced to Germany from North America.

The macroscopic findings of the present case, characterized by splenomegaly, ataxia, and jaundice, are consistent with observations described for AMDV infection in wild and domestic striped skunks [26,27]. Histopathological lesions observed in this study were dominated by multisystemic lymphoplasmacellular inflammation in various organs, including the liver, kidney, stomach, intestine, and central nervous system. Since lymphoplasmacellular inflammatory alterations in various organ systems were already reported in other species infected with amdoparvoviruses, the observed changes are probably directly related to the viral infection [5,10,23,26,27]. The fibrosis in the kidney and liver, the bile duct hyperplasia, and the hyperplasia detected in the spleen and lymph nodes are secondary to the inflammatory processes. Vascular lesions might have been induced by the deposition of antigen–antibody complexes in vessel walls resulting in vasculitis due to a type III hypersensitivity reaction as well as consecutive dystrophic calcification, insudation of lipids, and cholesterol crystal formation (atherosclerosis). Nevertheless, atherosclerosis might have developed independently from virus infection in the present case. Furthermore, calcifications in blood vessels and other organs can be caused by renal insufficiency due to the retention of phosphate, secondary hyperparathyroidism, and precipitation of mineral complexes on endothelia damaged by uremia. The present white matter spongiform degeneration (uremic encephalopathy) also represents a characteristic finding of uremia. The suppurative rhinitis has most likely been induced by secondary bacterial infection and represents an incidental finding similar to vacuolization of adrenal glands, extramedullary hematopoiesis in the spleen, and foam cell granulomas in the lung. Atelectasis, alveolar edema, and hemorrhages within the lung were interpreted as agonal changes.

For AMDV and related carnivore amdoparvoviruses, it has been demonstrated that the structural VP2 gene is genetically more conserved than the non-structural NS1 gene [7,21]. We were able to confirm that SKAV also shows increased conservation of the VP2 gene sequence. This may explain why we were unable to detect evidence of a recombination event in the genome of the German SKAV strain, despite the discordant geographical clustering observed in the NS1 and VP2 phylogenetic trees. Alternatively, the number of available SKAV full genome sequences may not currently be sufficient to enable the detection of a recombinant event in this strain. However, the observation that AMDV strains lack clear geographical clustering [12,35] could not be confirmed for SKAV. The better-defined geographical separation of subclades for SKAV sequences might be due to lower levels of artificial virus spread mediated by animal trading in contrast to the extensive worldwide spread of AMDV due to the movement of mink for the purpose of commercial fur farming activities.

SKAV has been previously shown to cross species barriers and infect American mink (*Neovison vison*) [18]. Based on this information and on the similarity to its close relative AMDV, SKAV can be assumed to be a potential threat to endangered carnivore species in Europe [36,37]. The establishment of endemicity of SKAV strains in wild species of mustelid species in Europe could be mediated by feral American minks that live in many European countries as an invasive species and have been proven to be susceptible to SKAV infection. Furthermore, due to the high mutation rate of carnivore amdoparvoviruses and the potential to recombine, more pathogenic variants could arise, or variants could become adapted to new species following host switches. Zoo animals present a special risk for the introduction of novel infectious diseases, as the close proximity between animals from different geographical areas facilitates interspecies virus transmission or transmission to reside wildlife species. Given the absence of previous reports documenting SKAV in wild mustelid species in Europe and underlined by the potential threat posed to endemic fauna, better controls and monitoring of the health status of imported mustelids should be considered to avoid the introduction of novel species of carnivore amdoparvovirus by acutely or persistently infected animals.

Knowledge of the diversity of amdoparvoviruses has been greatly expanded in recent years, with several new species reported and the host range being possibly extended to bats and rodents [38]. This highlights the necessity for increased surveillance of potential reservoir species and further investigations into the diversity of amdoparvoviruses. In summary, this study describes the first detection of SKAV outside North America, indicating the intercontinental spread of SKAV via the transportation of wildlife animals.

## Data Availability

The sequencing data presented in this study are available in GenBank, accession no. OQ294046.
